# 

**Published:** 1890-12-20

**Authors:** 


PRICE ONE PENNY.

No. 221, Vol. IX.—December 20th, 1890.

[Registered at the General Post Office as a Newspaper for transmission in the United Kingdom and Abroad.]

WITH EXTRA NURSING SUPPLEMENT.

Per Annum, 6s. 6d.; post free.

Monthly Parts, 6d.; post free 8d.

Ditto per Annum, 8s., post free.

### THE HOSPITAL

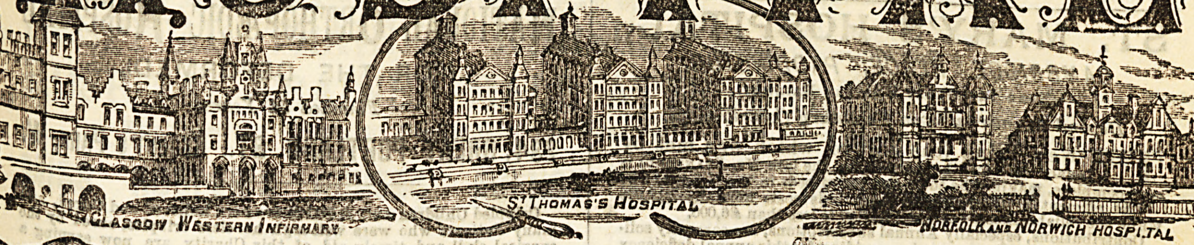

A WEEKLY JOURNAL OF
Science, Medicine, Nursing and Philanthropy.

SATURDAY, DECEMBER 20th, 1890.

The Poor Man's Christmas - 181

Passing Topics.—Hospitals from a Statistical Point of View - 182

The Doctor's Armchair.—"Charity's Much-injured Name" 183

Working Women in Large Towns.—XVIII.—Aids for Working Women (*Continued*) - 184

A Hospital Garden - 185

The History and Capabilities of Herbal Simples. By W. T. Fernie, M.D.—XXIV.—Cress - 186

Everybody's Page - 187

Notes and News - 188

Annotations.—"An Old Song"—American and English Girls—An Endowment for the Bideford Hospital - 190

The Practitioner's Mirror and Retrospect:— The Post-Graduate Course at the Brompton Consumption Hospital—The Physical Diagnosis of Diseases of the Lungs and Pleura - 191

The Convulsive Cough of Puberty. By Sir Andrew Clark, M.D, F.R.S. - 192

Sussex County Hospital - 193

Editor's Letter Box—Northerner *v.* Southerner - 195

Scraps and Gleanings - 1[illegible]5

The Student of Medicine.—Editorial Notes—Appointments—Notes from the Schools—Students' Medical Societies—Pass Lists—Athletics - 196

General Charities.

EXTRA SUPPLEMENT—THE NURSING MIRROR.

En Passant.—Rural Nursing Association—Ward Maids—Strong and True—A Nurse for Buxton—Short Items—The Fate of Reformers—Fever Hospitals - lix

Lectures on Surgical Ward Work and Nursing. By Alex. Miles, M.B. (Edin.), C.M., F.R.C.S.E. Lecture VII.—Preparing for an Ordinary Dressing - lx

Nursing Medals and Certificates. No. II.—The Charing Cross - lxi

For Reading to the Sick.—Preparing for Christmas - lxi

Nurses on Their Travels.—"A Winter Holiday" - lxii

Everybody's Opinion.—Asylum Attendants The Diet of Nurses—Attendants at Berry Wood, lxii; A Wedding Gift—Nurses' Hours - lxiii

Examination Questions—Notes and Queries - lxiii

Off Duty—On £30 a-year - lxiv

Appointments - lxiv

To "The Hospital" Supplement - lxiv

Amusements and Relaxation - lxiv

